# Impact of Trophic Mode-Driven *Chlorella* Biomass on Vegan Food Emulsions: Exploring Structure and Functionality

**DOI:** 10.3390/molecules30040766

**Published:** 2025-02-07

**Authors:** Sheyma Khemiri, Albano Joel Santos, Anabela Raymundo

**Affiliations:** LEAF-Linking Landscape, Environment, Agriculture and Food Research Center, Associate Laboratory TERRA, Instituto Superior de Agronomia, Universidade de Lisboa, Tapada da Ajuda, 1349-017 Lisboa, Portugal; joelsantos@isa.ulisboa.pt (A.J.S.); anabraymundo@isa.ulisboa.pt (A.R.)

**Keywords:** *Chlorella*, emulsion, vegan product, viscoelastic properties, antioxidants, vitamin B12

## Abstract

Aligning with sustainable green practices, this study examines the partial replacement of chickpea protein isolate with commercially available autotrophic *Chlorella vulgaris* (Auto-*Chlorella*) and heterotrophic *Parachlorella kessleri* (Hetero-*Chlorella*) to assess impacts on food emulsions’ properties and potential functional value. Rheology and texture analysis show that *Chlorella* biocompounds enhance emulsions by creating a synergistic network with chickpea proteins. The type of *Chlorella* used significantly influences emulsion characteristics due to differences in culture and processing conditions. Hetero-*Chlorella* contributed to more structured emulsions, revealed by higher values of the viscoelastic functions (G′, G″, and G^0^_N_), indicating a complex three-dimensional network (*p* < 0.05), while Auto-*Chlorella* excelled in augmenting dietary elements (*p* < 0.05), leading to emulsions rich in antioxidants and allowing for a ’rich in iron’ claim. Both types contribute to smaller oil droplet size, improved firmness, adhesiveness, and appealing coloration (*p* < 0.05). Preliminary findings on Vitamin B12 content suggest promising bioavailability potential. However, the nutritional density of *Chlorella* emphasizes the need for careful microbiological stability. Produced on a lab scale without preservatives, these emulsions highlight the need for preservation strategies in large-scale production. This research supports the potential for industrial microalgae-based mayonnaise, addressing consumer demand for innovation while prioritizing safety.

## 1. Introduction

Protein and water, alongside fat, carbohydrates, vitamins, and minerals, constitute fundamental components of food. The physical incompatibility between fat and water, being mutually insoluble, is a well-known phenomenon. However, emulsions can disperse both fats and water, creating a macroscopically homogeneous state—a colloid.

Due to the energetically unfavorable interaction between fat and water, emulsions are thermodynamically unstable and require high mechanical energy for dispersion. However, these dispersions are not considered true emulsions unless they exhibit sufficient stability to remain dispersed for a reasonable amount of time, which may vary from a few seconds to several years based on their intended use. Ensuring stability for an extended period is particularly crucial in the case of food emulsions, where stability over months is an important demand [1]. The energy input necessary for emulsion formation can be minimized through the use of emulsifiers. These emulsifiers, often amphiphilic compounds with relatively low molar mass, can swiftly assemble at the interface where droplets form during emulsion mixing [2].

Traditionally, the stabilization of oil-in-water food emulsions has relied on egg proteins, particularly lipoproteins found in the yolk, which constitute approximately 31% of egg protein and are highly effective as an emulsifier [3]. Eggs play a crucial role in ensuring both the kinetic stability of emulsions and their sensory attributes, including taste and color. However, a variety of other proteins, such as soy, whey, and casein, have also been used as emulsifying agents in different parts of the world, reflecting diverse culinary practices and ingredient availability. In recent years, rising concerns about allergies and cholesterol-related diseases, coupled with the increasing demand for “clean-label” and “plant-based” foods, have driven consumer interest in exploring alternative stabilizers [4,5,6].

A notable surge in the adoption of vegan diets has been observed recently, with a growing preference for plant-based food sources. While some individuals have embraced veganism due to concerns about animal cruelty and adherence to regional ethical practices. Such diets might fall short in providing essential nutrients, including amino acids and vitamins. For instance, some vegan and/or vegetarian diets may struggle to meet the daily requirements of vitamin B12 for physiological needs [7]. Additionally, cereals may lack lysine, and legumes might have low levels of sulfur-containing amino acids, such as methionine and cysteine [8].

Rich in proteins, chlorophyll, lipids, and polysaccharides, *Chlorella* biomass is valued for its nutritional properties and functional versatility [9,10]. Its natural emulsifying properties make it particularly attractive for clean-label food formulations and sustainable applications, offering advantages over synthetic emulsifiers.

In previous studies, significant progress was made in exploring the potential of microalgae as ingredients in food emulsions. Batista and coworkers [11] focused on the effects of lutein and phycocyanin, specific pigments derived from microalgae, on the rheological and textural properties of pea protein-stabilized emulsions. Their research demonstrated how the inclusion of these pigments could influence the properties of emulsions but was specifically limited to the effects of isolated molecules. Raymundo and coworkers [12] used *Chlorella vulgaris* biomass as a fat mimetic and emulsifier, showing its ability to replace oil in emulsions, with varying performance between different microalgal forms. In contrast, Gouveia et al. [13] utilized microalgae primarily as natural colorants, with additional benefits such as antioxidant activity and improved oxidation resistance. While these studies demonstrated the potential of microalgae to enhance emulsion properties, they primarily focused on individual components like pigments or oils and used relatively low concentrations (0.2–2%) of microalgae.

In our current work, we take a different approach by using the entire biomass of *Chlorella vulgaris* and *Parachlorella kessleri* to replace chickpea protein isolate in emulsions. This novel approach leverages the full spectrum of bioactive molecules present in the biomass, avoiding the need for extraction methods focused on specific compounds, as seen in previous studies. We also explore the use of higher microalgae concentrations (4%), addressing a gap in earlier research where concentrations typically ranged from 0.2% to 2%. Furthermore, we examine the impact of two commercially available *Chlorella* species, cultivated under different conditions—autotrophically and heterotrophically.

Microalgae are known to respond dynamically to environmental changes, often accumulating specific molecules as a strategy for adaptation. This responsiveness can be tested at the laboratory scale by imposing specific cultivation conditions, such as applying different trophic modes—autotrophy, heterotrophy, or mixotrophy—or through nutrient depletion, which can enhance the bioaccumulation of targeted compounds [14]. These findings reflect the distinctive features and composition of microalgae as functional ingredients, emphasizing the importance of ongoing research, such as the present work, to advance the application of these sustainable resources. By highlighting the effects of different cultivation modes on the physical and biochemical characteristics of the microalgae, we aim to deepen the understanding of how these variations influence the functionality of microalgae-based food emulsions. This work represents an important step toward advancing the application of microalgae in food products, offering insights that could broaden the use of microalgae biomass while ensuring scalability for industrial production, especially as these species are commercially available in large quantities.

## 2. Results and Discussion

### 2.1. Composition of Microalgae Biomasses

The two *Chlorella* microalgae biomasses used in this study underwent biochemical characterization under identical conditions to differentiate the impact of the applied trophic modes on macromolecular levels. The results were compared with existing literature to further emphasize the effects of cultivation conditions on various species of microalgae, or even within the same strain. As shown in Table 1, both *Chlorella* biomasses exhibited a suitable composition for food enrichment, with dry matter—encompassing protein, carbohydrates (including fiber), and lipids—constituting the major component in both cases. Notably, significant differences were observed amongst the trophic modes. The literature extensively reports the influence of culture conditions, particularly the trophic modes applied to microalgae, on their biochemical profiles. Table 1 clearly illustrates how microalgae metabolize compounds under varying cultivation conditions. For instance, Sharma and coauthors [15] discussed the effects of autotrophic, mixotrophic, and heterotrophic growth on total protein, lipid, and carbohydrate levels in *Parachlorella kessleri*, noting that mixotrophic conditions yielded the highest protein content, while autotrophic conditions produced the lowest. In this current study, protein emerged as the most abundant component, with Auto-*Chlorella* containing the highest protein level at 51.46%. However, the combined carbohydrate (12.21%) and fiber (18.14%) content was greater in Hetero-*Chlorella*, highlighting the complex interplay between trophic modes and biochemical composition in microalgae.

### 2.2. Rheological Properties

#### 2.2.1. Dynamic Oscillatory Tests

**Figure 1:**
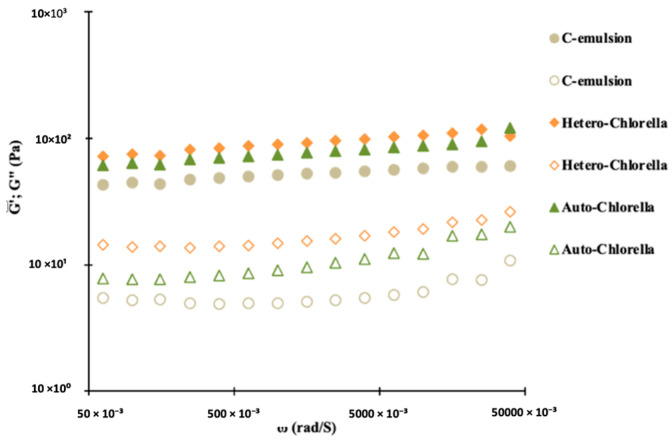
Viscoelastic response of microalgae-based emulsions. G′ (elastic component—filled symbol), G″ (viscous component—open symbol). C-emulsion is the product without *Chlorella* addition.

The viscoelastic properties of the chickpea-based emulsions with 4% of whole *chlorella* biomass can be observed in Figure 1, showcasing variations in the viscoelastic functions (G′ and G″) with frequency. These emulsions exhibit mechanical spectra typical of protein-stabilized emulsions, where an elastic network develops due to extensive bridging flocculation processes, e.g., [12]. In the studied frequency range, the storage modulus (G′) consistently surpasses the loss modulus (G″), and the evolution of G′ with frequency suggests the development of a plateau region. The plateau modulus G^0^_N_, studied as a characteristic parameter of this region, is depicted in Table 2.

Dynamic measurements reveal that replacing 4% of chickpea protein isolate with microalgae biomass reinforces the emulsion structure. This is evidenced by a significant increase in the plateau modulus G^0^_N_, with the Hetero-*Chlorella* emulsion exhibiting the strongest network (Table 2). These results can be cross-referenced with G′ values at a specific frequency (1 Hz) (Table 2). This finding supports the potential use of microalgae material as a protein emulsifier, along with potential advantages as a coloring and antioxidant agent, which will be further discussed in the following section. Potential interactions between chickpea protein and microalgal biomass could further enhance the reinforcement of the emulsion structure by fostering the formation of physical entanglements [17]. This reinforcement is more pronounced when the Hetero-*Chlorella* biomass is used (the yellow emulsion). In addition to differences in protein content between the two *Chlorella* biomasses used (Table 1), variations are also likely attributed to distinctions in cell wall characteristics. A recent study by [18] thoroughly examined the effects of three trophic modes (autotrophic, heterotrophic, and mixotrophic) on growth parameters, productivity of individual cell components, and biochemical composition of several *Chlorella* species. Special attention was given to protein profiles identified through SDS-PAGE and two-dimensional gel electrophoresis combined with MALDI-TOF/TOF MS. The study concluded that the qualitative differences in protein profiles were attributable to the culture conditions rather than the species used. For a more in-depth exploration of the potential of microalgae proteins as a green alternative for stabilizing fluid interfaces and emulsion formation, readers are referred to the recent review by [19].

On the other hand, carbohydrates represent another significant component of the *Chlorella* biomasses used (Table 1). Although the total carbohydrate content has been primarily measured, it is crucial to distinguish between storage carbohydrates and structural carbohydrates (i.e., cell wall-related polysaccharides), as their presence could potentially impact the rheological properties of the enriched food products [20]. One approach to leverage these structural biopolymers is to isolate the cell wall-related polysaccharides for use as food hydrocolloids. Similarly, while fractionation and protein isolation enhance functionality, each purification step reduces absolute protein yield, making the final product more resource intensive.

Incorporating the entire biomass, as in this study, offers a more appealing approach by eliminating the need for extraction solvents, aligning closely with more sustainable principles. One strategy to mitigate associated costs involves valorizing multiple microalgae constituents and by-products, such as lipids, pigments, or vitamins. For example, Braga and coauthors [4] demonstrated that removing valuable protein pigments (C-phycocyanin) did not hinder the emulsification properties of *Spirulina* residual biomass, enabling the formulation of stable protein-rich emulsions. In some cases, less purified extracts may outperform more purified isolates. Another instance involves polysaccharides in the protein isolate from *Tetraselmis* sp., enhancing emulsion and foam stability, as discussed by [21]. Regarding the emulsions produced in this study, visual stability persisted for up to 60 days of storage (last day of microbial stability assessment). Viscoelastic properties and particle size distribution were measured only on day 1, as these analyses were intended to characterize the emulsions’ initial structural properties. Changes in these parameters are typically gradual under refrigerated storage and unlikely to occur within the short storage periods studied. Moreover, if there were any significant changes in these properties immediately after production, it would point to a fundamental instability in the formulation, which was not observed.

Future work will expand on this baseline, developing a vegan mayonnaise and comprehensively studying the formulation. In general, the prevalence of elastic behavior over viscous characteristics, along with the weak gel formation phenomenon, has been reported previously in plant-based emulsions [5,22]. Raymundo and coauthors [12] produced emulsions incorporating pea protein and *Chlorella vulgaris*, and the plateau modulus G^0^_N_ was notably higher than in the standard emulsions. Similarly, ref. [11] examined the effects of pigment addition on the rheological behavior and microstructure of emulsions. Their findings indicated that the phycocyanin emulsion exhibited significantly higher values (*p* < 0.05) for viscoelastic functions (G′, G″, and G^0^_N_) compared to the control, suggesting a more complex three-dimensional structure.

#### 2.2.2. Steady-State Slow Curves

**Figure 2:**
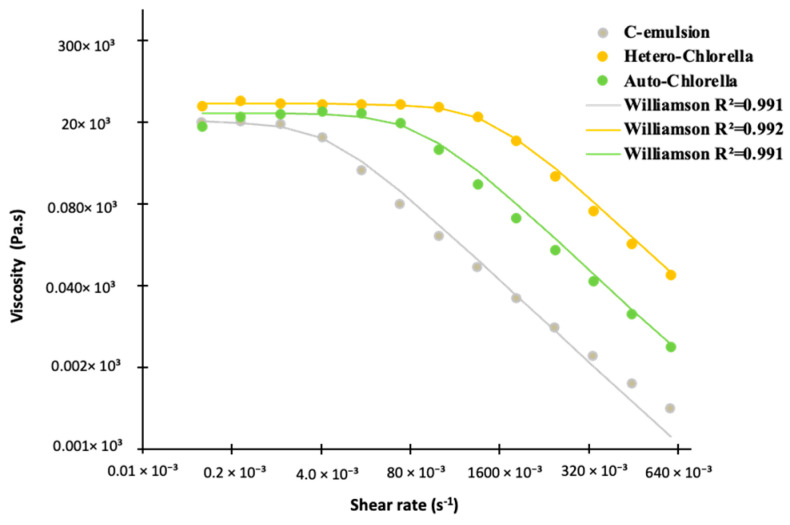
Variation in apparent viscosity (η) as a function of shear rate (γ) of microalgae-based emulsions. C-emulsion is the product without *Chlorella* addition. Lines represent Williamson’s model adjustment.

Figure 2 depicts the variation in apparent viscosity with shear rate. All samples exhibit shear-thinning behavior, suggesting a potential structural degradation during emulsion shearing. This behavior is indicative of the deformation of aggregated three-dimensional oil droplet networks under shear, resulting in lower viscosity. This effect is notably more pronounced in the control curve (chickpea-based emulsion).

Additionally, Table 3 presents the outcomes of the flow behavior test for emulsion samples, fitted with Williamson’s model (0.991 < R2 < 0.992). The flow behavior index (m) of the microalgae-containing samples does not demonstrate significant differences compared to the control sample, predominantly indicating a shear-thinning behavior (m < 1). The consistency coefficient (k), associated with viscosity at low shear rates, peaks in the sample containing Auto-*Chlorella* (the green emulsion). The difference between the control sample and the microalgae-based emulsion is not statistically significant (*p* > 0.05). Furthermore, the zero shear limiting viscosity (η_0_) shows a meaningful increase (*p* < 0.05) with the addition of microalgae supplementation (Table 3). This observation holds prominent implications for the food industry, as apparent viscosity emerges as a crucial quality control parameter during various technological processes, such as mixing, pumping, transportation, and storage. Moreover, it plays a pivotal role in influencing consumer acceptance [23]. Comparing the Williamson parameter values from our current study with those from Braga and coworkers [4], who used non-animal proteins to formulate emulsions with varying ratios of chickpea and *Spirulina* residual biomass (100:0, 75:25, 50:50, 25:75), all the apparent viscosity values were lower than those obtained in our study.

### 2.3. Droplet Size Distribution

**Figure 3:**
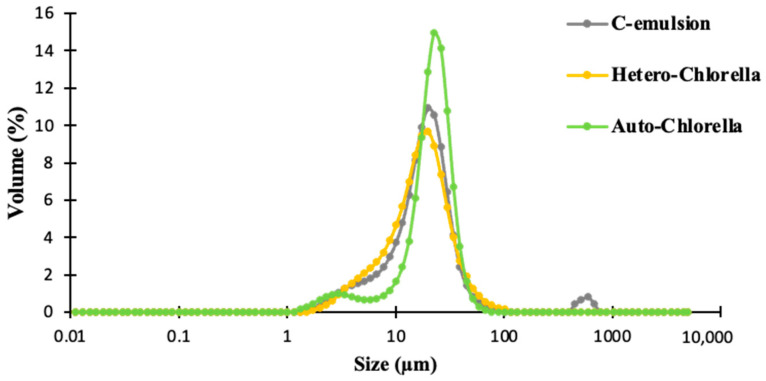
Comparison of droplet size distribution of microalgae-based emulsions. C-emulsion is the product without *Chlorella* addition.

**Figure 4:**
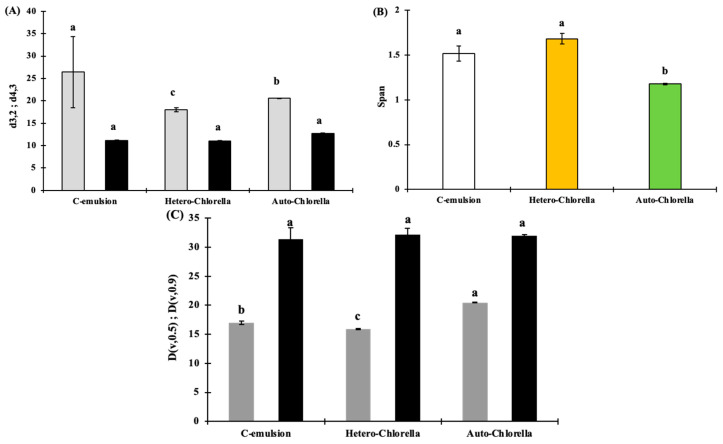
Span parameter (**B**), De Brouckere diameter (d4,3) (grey), and Sauter diameter (d3,2) (black) (**A**), and median diameter (D0.5) (grey) and percentile diameter (D0.9) (black) (**C**) from droplet size distribution of microalgae-based emulsions. C-emulsion is the product without *Chlorella* addition. Different letters in each bar indicate significant differences (*p* < 0.05).

Figure 3 and Figure 4 illustrate the results of particle size distribution analysis conducted on emulsion samples. The blank sample consisting solely of chickpea protein exhibited the largest mean particle size, with a d4,3 value of 26.43 µm. Conversely, the sample containing 4% Hetero-*Chlorella* demonstrated the smallest mean particle size, measuring 18.02 µm.

The De Brouckere parameter (d4,3) combines information from both the larger particles and the smaller particles. This indicates a higher polydispersity level for oil droplets in the control sample compared with microalgae-emulsion samples. Higher polydispersity levels in emulsions have been linked to increased instability, including phenomena such as creaming and Ostwald ripening [19]. While these phenomena were not directly observed in the current study, the blank sample exhibited the lowest physical stability when compared to the other samples, suggesting a potential for greater instability under longer storage periods or different conditions. Conversely, the span values amongst the samples remain relatively consistent (*p* > 0.05), suggesting that the majority of particles in all samples are concentrated within a specific size range.

The d3,2 (volume-weighted mean diameter) and d4,3 (De Brouckere diameter) provide distinct insights into the particle distribution. The d3,2 primarily focuses on the average size based on the volume of droplets, which may be influenced more by the smaller particles in the distribution. The lack of significant change in the d3,2 value indicates that the average particle size based on volume distribution remained relatively stable across the samples.

Analysis of the particle size distribution curve (Figure 3) reveals that the emulsion containing the green Auto-*Chlorella* exhibits the highest peak, indicating a greater proportion of larger oil droplets dispersed within the aqueous phase compared to the other samples. Additionally, both the control and Auto-*Chlorella* emulsion curves display small additional peaks or shoulders, indicative of a bimodal distribution. These features typically represent subsets of particles within the sample that possess slightly different sizes compared to the majority. A unimodal curve was obtained for the emulsion with Hetero-*Chlorella*, which exhibited the lowest d4,3 value. Some positive characteristics documented for this specific Hetero-*Chlorella* include resistance to shear stress, poor adhesion to bioreactor surfaces, and a low tendency to form aggregates [24]. These traits were reflected in the resulting enriched emulsion (with Hetero-*Chlorella*).

The lower d4,3 value suggests that, on average, the particle sizes in this sample are smaller compared to the other samples. Such a distribution typically indicates greater stability, a notion supported by the preceding emulsion rheology results. The smallest particle diameter was achieved by Ebert et al., (2019) in their emulsion prepared by *Chlorella sorokoniana* using high-pressure homogenization. They found that a 1.0% concentration of soluble protein from *Chlorella sorokiniana* resulted in a monomodal droplet size distribution with a small volume (d4,3 = 232.22 nm).

For a more comprehensive description of the particle size distribution, we also examined the D0.5 and D0.9 values, which provide insight into the median and larger end of the particle size distributions, respectively. The d0.5 values were 17 µm for the control, 15.87 µm for the Hetero-*Chlorella* emulsion, and 20.46 µm for the Auto-*Chlorella* emulsion, suggesting that the control emulsion has a slightly smaller median particle size compared to the Hetero-*Chlorella* emulsion but larger than the Auto-*Chlorella* emulsion. As for the D0.9 values, which represent the 90th percentile of the particle size distribution, the control emulsion showed a value of 31.34 µm, while both microalgae-based emulsions showed a D0.9 value of 32 µm.

The stability of a system, whether it’s an emulsion or another colloidal system, can be influenced by various factors, including the size of the particles present [4,11]. However, whether larger or smaller particles enhance stability depends on the specific situation and how particles interact with their surroundings. Smaller particles often have a higher surface area-to-volume ratio, which allows for stronger interactions with stabilizing agents or the surrounding medium, ultimately improving stability. While this study primarily focused on particle size distribution, measuring zeta potential could offer additional insight into the emulsion’s stability by assessing its resistance to destabilization phenomena such as creaming, sedimentation, flocculation, and coalescence. This aspect could be explored in future studies to complement the current findings.

### 2.4. Texture Profile

**Figure 5:**
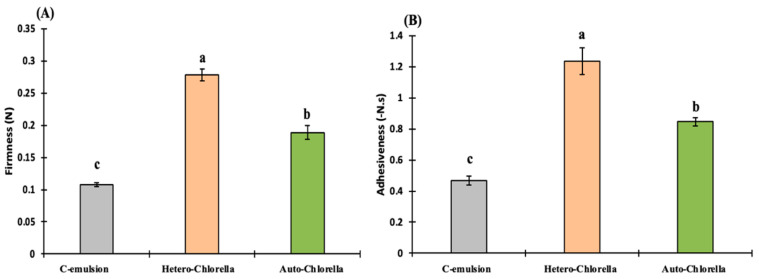
Texture profile of microalgae-based emulsions. C-emulsion is the product without *Chlorella* addition. Different letters in each bar for each parameter indicate significant differences (*p* < 0.05).

**Figure 6:**
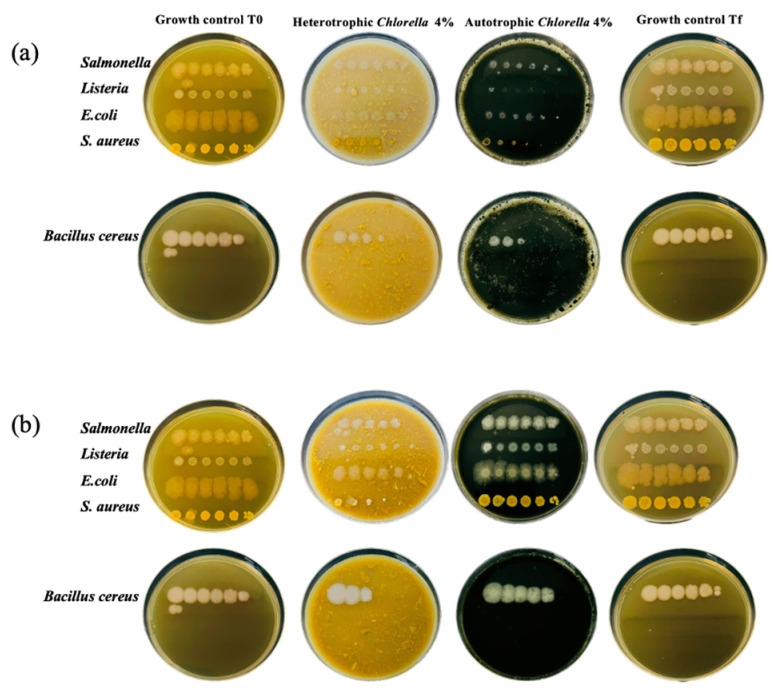
Plates resulting from in vitro antimicrobial activity determination for pathogenic microorganisms, incubated for 48 h at adequate temperatures. The growth control is BHI medium. From left to right, drops from 10^−1^ to 10^−5^ dilutions. (**a**) Results for the preliminary test with no nutritive media, only the microalgae biomass as substrate. (**b**) Test with nutritive media and microalgae biomass.

Figure 5 illustrates the texture parameters, namely firmness and adhesiveness, of the emulsion samples. Firmness exhibited a consistent increase with the addition of microalgae biomass, and this observed increment, with a precision value exceeding 95%, was statistically significant (*p* < 0.05). For instance, the Hetero-*Chlorella* emulsion required a notably higher force for deformation (0.27 N for firmness and 1.23 N for adhesiveness) compared to the other samples, indicating a greater resistance to spread in the texture (Figure 6). Based on the penetration test results, it can be concluded that the microalgae-based emulsion samples may not perform as similarly as the control sample in domestic or commercial applications. This conclusion aligns with both the macroscopic observation of the samples during handling and the viscoelastic findings mentioned earlier, which indicated that the control emulsion exhibited a more fluid-like consistency compared to the semi-solid state observed in the microalgae-based samples. Additionally, previous studies by [25,26] have highlighted that the firmness of emulsions is negatively influenced by the size of dispersed oil droplets. As depicted in Figure 3, emulsions with larger droplet sizes (such as the emulsion with green Auto-*Chlorella*) and/or higher levels of polydispersity (like the control emulsion) tend to exhibit lower firmness and adhesiveness. Even though microalgae biomass served as a protein substitute, it remains challenging to definitively ascertain whether the primary determinant of the texture and rheological properties of the samples was the total protein content, the specific types of proteins, or a combination of both factors. The precise composition and structure of proteins in microalgae remain unclear and can vary significantly depending on various factors, such as growth conditions and physiological states [18].

In a prior investigation conducted by [27], which examined the sensory and texture attributes of mayonnaise samples prepared with varying emulsification intensities and, consequently, droplet sizes, it was observed that higher emulsification intensities and smaller droplet sizes yielded mayonnaise samples that exhibited significantly greater firmness and adhesiveness. In the current study, all emulsion samples were generated by using the same food processor set at a consistent speed and within a uniform total processing period. Therefore, any alterations noted in texture and oil droplet size are not expected to be a consequence of instrumental emulsification intensity. It is probable that the elevated concentration of the emulsifying agent in both microalgae biomasses (comprising proteins, polysaccharides, lipids, and other things) played a role in reducing oil droplet size and enhancing firmness and adhesiveness.

Additional research is required to enhance our understanding of this process, potentially through comparisons of various concentrations of the same ingredient and their impact on texture, or by standardizing, for instance, the total protein and/or carbohydrate contents in the final recipe as one of the controlled variables when comparing all ingredients.

### 2.5. Color Parameters and pH of the Emulsions

Consumers’ sensory acceptance is strongly influenced by the appearance of food products, with color being one of the most critical attributes to consider. Table 4 presents the color, ∆E* values, and pH measurements of the emulsion samples at initial stages and after 15 days, reflecting a focus on capturing early-stage changes in these parameters, which are crucial indicators of the emulsion’s visual and chemical stability during initial storage.

Regarding the pH of the emulsions, a noticeable difference was observed between those formulated with autotrophic and heterotrophic microalgae, as well as the control emulsion. The control emulsion, which did not contain any microalgae, exhibited the highest pH, with a value of 6.83 on day 1, and remained relatively stable after 15 days, decreasing slightly to 6.77. The Auto-*Chlorella* emulsion exhibited a relatively higher pH compared to the Hetero-*Chlorella* emulsion, with the latter showing a more acidic pH after 15 days. This difference can be attributed to the metabolic processes of the microalgae; heterotrophic growth relies on organic carbon sources, often leading to the accumulation of organic acids, contributing to a lower pH.

The L* (lightness) parameter of the emulsions significantly impacts the product’s appearance. The highest L* value (75.05) amongst the samples, following the control sample (L* 89.55), was observed in the Hetero-*Chlorella* emulsion. All formulations exhibited negative a* values (Table 4). As anticipated, the formulation with the green Auto-*Chlorella* exhibited significantly higher values (*p* < 0.05) for the a* modulus, attributed to the high chlorophyll content in this biomass, as demonstrated in previous research utilizing the same strain of *Chlorella* [28]. Nevertheless, the blackish green color seen for the Auto-*Chlorella* emulsion was becoming lighter with time (*p* < 0.05). A similar trend was demonstrated in a previous work using *Hematoccocus pluvialis* and *Chlorella vulgaris* in emulsions, where a significant (*p* < 0.05) decrease in the a∗ value over time was observed, revealing the existence of color instability [13]. Additionally, all emulsions are distinguished by the naked eye when compared to the control emulsion, since ∆E* values are greater than 5. Color instability in emulsions formulated with microalgae biomass rich in chlorophyll may be influenced by light exposure. While the emulsions in this study were stored in glass containers, which could have contributed to color degradation, we did not include additional experiments to assess the impact of light-blocking packaging materials or antioxidants. Therefore, future studies should explore the use of opaque packaging or natural antioxidants to prevent color degradation and further evaluate the effects of storage conditions on color stability in microalgal emulsions. In all instances, the darkening observed following the addition of microalgae does not detract from the appeal of the emulsions, as consumers are accustomed to purchasing products rich in vegetables, often associating darker colors with such products. Concerning the b* parameter, all samples still exhibit yellow tones (Table 4). These values could potentially be diminished, as the predominant yellowness in emulsions typically arises from egg yolk and oil [22]. However, the Hetero-*Chlorella* emulsion presented a visually striking yellow color, notably more pronounced than the other samples examined in this study (*p* < 0.05). This heightened color intensity may be attributed to the higher concentration of carotenoids present in this microalgae biomass.

### 2.6. Bioactivity of Emulsions

The antioxidant potential, as a notable biological effect, is also a key feature emphasized in this work. The inclusion of vegetable oils, comprising 65% of the current formulation, in food emulsions can pose technological challenges due to their high susceptibility to oxidation. One commonly employed strategy to enhance the oxidative stability of emulsion systems involves the incorporation of antioxidants [29]. Antioxidants have the potential to be distributed across various physical compartments within a conventional oil-in-water emulsion. Hydrophilic components can be dispersed within the external water phase, while lipophilic components can be integrated into oil droplets within the oil phase. When using microalgae rich in antioxidants, these bioactive compounds can also be introduced into the emulsion, potentially contributing to its overall antioxidant capacity. Furthermore, microalgae contain molecules with different polarities [30], which could accordingly be dispersed in the different compartments and enhance the overall antioxidant ability of the emulsion. Nevertheless, it’s important to carefully consider the composition of the emulsion and potential interactions between its components and the assay reagents to ensure accurate and reliable assessment of antioxidant activity. The methodology employed by the authors is based on phase separation, as described in the Section 3. Separating the organic phase from the emulsion before conducting antioxidant assays enables the removal of potential lipid oxidation products generated during storage or processing, which could interfere with the assay process. This approach provides a cleaner matrix for assessing the efficacy of exogenously added antioxidants without the confounding effects of lipid oxidation.

All samples exhibited antioxidant activity, which was assessed using two methods alongside the determination of total phenolic compounds (PC). Microalgae-emulsion samples demonstrated notably higher scavenging and reducing abilities, with a significant contrast observed between the two biomasses employed (Table 5). Notably, *Chlorella* is recognized as a significant source of bioactive compounds, exhibiting noteworthy antioxidant properties [10,15]. The composition of macro and micronutrients in algae can vary as a function of the cultivation conditions, harvesting processes, and the type of technological processing utilized. Consequently, these factors can influence the bioactive capacity of algae-containing products. The enhancement in bioactive content observed in supplemented emulsions, notably indicated by higher phenolic compound (PC) levels, resulted in a significant improvement in the antioxidant activity of these samples (*p* < 0.05) (Table 5). Specifically, the emulsion containing Hetero-*Chlorella*, with the highest PC content (109.21 µg GAEq/g), exhibited elevated DPPH (85.78 µg TroloxEq/g) and FRAP (31.38 µg TroloxEq/g) antioxidant capacities.

### 2.7. Content of Essential Dietary Elements

In this study, essential minerals were accurately determined and quantified, highlighting the nutritional enhancement achieved by incorporating microalgae biomass to replace 4% of the chickpea protein in the formulation (Table 6). While the levels of magnesium, iron, zinc, and calcium in the emulsions increased significantly compared to the control emulsion, it is important to note that these increases are below the threshold typically required to classify a product as “enriched” by regulatory standards. Specifically, calcium, a crucial nutrient for bone health and muscle function, increased by over 100% in the emulsion with Auto-*Chlorella*.

On the other hand, iron, which is essential for the formation of red blood cells and the transportation of oxygen throughout the body, increased seven-fold, reaching a level that allows the emulsion to carry the “rich in iron” nutritional allegation. This enhancement in iron content is vital, considering its role in energy metabolism and cognitive development [31]. Emulsions serve as the foundation for more complex food products such as mayonnaise and salad dressings, which are consumed worldwide. Therefore, creating an improved version with enhanced nutritional values represents a significant advancement. Regarding vitamin B12, which is crucial for brain health, DNA production, and red blood cell formation [32], this study builds upon previous investigations by the authors into its quantification within microalgae biomass. These earlier efforts [33] have laid the groundwork for understanding vitamin B12’s availability in microalgae, underscoring the significance of this nutrient in enhancing the nutritional profile of algae-based food products. The challenge lies in quantifying this critical vitamin in food products, especially plant-based ones, to address the deficiencies commonly faced by vegetarians and vegans.

Applying the methodologies described in the Section 3, we detected 0.5 mg of vitamin B12 per 100 g of emulsion (with Auto-*Chlorella*), an encouraging outcome considering the nutritional labeling and potential health benefits. This biomass was anticipated to contribute beneficially, as indicated by the label from the production company, which showed a level of 1036 μg of vitamin B12 per 100 g. However, determining the appropriate amount of microalgae biomass to use was crucial to enhancing the nutritional profile of the enriched food product and meeting consumer expectations. Furthermore, it is critical to consider the variability that can occur with the use of distinct analytical methods, alongside the sensitivity and detection limits of the equipment. To ensure consistency and accuracy, the microalgae biomasses as well as the emulsions were analyzed under uniform HPLC conditions, as detailed in [33]. It’s worth noting that the recommended daily intake of B12 for an average adult is about 2.4 μg, suggesting that 1 g of Auto-*Chlorella* emulsion could significantly contribute to daily nutritional requirements. However, another aspect to consider is the bioaccessibility and bioavailability of cobalamin in microalgae and, by extension, in microalgae-based products. Although not the focus of the current research, literature indicates that cobalamin bioaccessibility and bioavailability in microalgae may be limited, with pseudocobalamin (inactive forms) being prevalent [31]. Yet, *Chlorella*-based cobalamin is reported to contain significant portions of active cobalamin, offering a promising avenue for nutritional enhancement in plant-based diets. Future studies will focus on investigating the bioavailability and bioaccessibility of cobalamin in microalgae-based emulsions, particularly how the matrix and processing conditions influence the release and absorption of this essential vitamin. This will help clarify the potential of *Chlorella* as a sustainable source of bioavailable Vitamin B12 in food formulations.

### 2.8. In Vitro Antimicrobial Activity

The in vitro antimicrobial activity of the two *Chlorella* biomasses was assessed through two phases of testing: Test I, aimed at evaluating the ability of pathogens to grow in the presence of microalgae; and Test II, designed to examine the microbial growth inhibitory capacity of microalgae when all necessary nutrients are present in a proper nutritive medium. In Test I, conducted without a nutritive medium, pathogenic bacteria displayed poor growth with observable difficulty in comparison to the control (Figure 6a), most likely due to a lack of sufficient nutrients. However, the slight growth of the pathogens, especially at the highest concentrations, cannot be denied. Microalgae, like other organic materials, contain various nutrients such as carbohydrates, proteins, lipids, and vitamins, which can serve as a food source for bacteria. When the microalgae biomass was mixed with agar to create a growth medium and inoculated with pathogenic bacteria, the bacteria were able to utilize the nutrients released from the microalgae, thereby proliferating. The preparation of the medium involved a sterilization step. Autoclaving entails subjecting the medium to high temperatures and pressures, which can lead to the breakdown of complex molecules present in the microalgae biomass. This breakdown could potentially release additional nutrients into the medium, making them more available for bacterial growth. Consequently, bacteria may have access to a richer nutrient source, promoting their moderate proliferation. On the other hand, in the nutritive medium plates (Figure 6b), the growth of all pathogens was similar to that of the positive control without microalgae addition. Nonetheless, a slight decrease in growth was perceived in the plate with Hetero-*Chlorella*. Hence, it is concluded that the tested concentrations of microalgae are not inhibitory to bacterial pathogens, although they do not constitute a significant source of nutrients. While microalgae are known to display antibacterial properties against certain bacteria, it is possible that the concentrations of microalgae used in our experiment (4% in the media), or these specific species of *Chlorella*, were not sufficient to effectively inhibit the growth of these particular pathogens. Indeed, the literature often reports on the antibacterial properties of microalgae, as they can produce various bioactive compounds such as peptides, fatty acids, and pigments that exhibit antimicrobial activity against certain bacteria [34]. However, the high temperatures and pressures during autoclaving could potentially impact the general stability and activity of bioactive compounds within the microalgae biomass [35]. These compounds may exhibit sensitivity to heat and pressure, leading to denaturation or degradation. Consequently, the antimicrobial properties of the microalgae may be compromised, potentially allowing bacteria to proliferate unchecked. Therefore, encountering scenarios where certain bacteria grow in the presence of microalgae, despite their known antibacterial properties, is a possibility that researchers may encounter and further investigate to comprehend the underlying mechanisms, particularly concerning their application in food products.

### 2.9. Microbial Stability of Emulsion

Microbiological evaluations of microalgae-based emulsions, formulated without the inclusion of any preservatives, were meticulously carried out. These analyses took place initially seven days following the production of the emulsions and were repeated after a period of 60 days, during which the samples were stored at a temperature of 4 °C. Our approach builds on previous work from our group, where evaluations were performed immediately after emulsion production and 30 days later under refrigerated storage at 4 °C [5]. In this study, we extended the timeframe to include assessments at 7 days and 60 days, aiming to explore the potential for longer-term microbiological stability.

The results, as detailed in Table 7, encompassed the enumeration of total mesophilic bacteria, fungi (including yeasts and molds), lactic acid bacteria, and E. coli, alongside the assessment of L. monocytogenes and *Salmonella* sp. In all examined samples, the counts for both *Listeria* and *Salmonella* were found to be below 10^2^ CFU/mL, a result that aligns with the satisfactory thresholds as per the standards set by the Instituto Nacional de Saúde Doutor Ricardo Jorge (INSA), Portugal’s esteemed national health laboratory [36]. However, it was observed that all samples displayed unsatisfactory levels of *Staphylococcus aureus*. This finding echoes the observations of [6], who noted that even the addition of potassium sorbate in standard mayonnaise preparations failed to inhibit the proliferation of this bacterium, suggesting that the underlying issue might be attributed to lapses in maintaining rigorous hygiene protocols during the production process. Over the course of 60 days of storage, a notable increase in the populations of lactic acid bacteria was recorded, whereas both fungi and total mesophilic bacteria populations experienced a decline. This particular dynamic may be indicative of the nutritional resources within the emulsions becoming increasingly scarce over time, leading to a decrease in viability and/or cultivability for certain microbial groups. This trend underlines the complex interplay between microbial communities within food matrices and highlights the importance of ongoing monitoring and optimization of food preservation strategies to ensure safety and extended shelf-life periods.

## 3. Materials and Methods

### 3.1. Microalgae Biomass Production and Characterization

The *Chlorella vulgaris* biomass was supplied by Allmicroalgae Natural Products (Pataias, Portugal). The supplier cultivated the biomass under autotrophic conditions using an adjusted version of Guillard’s F2 medium as the growth medium. The *Parachlorella kesslerii* biomass (purchased from Algaeforfood) was cultivated heterotrophically in a bench-top fermenter operated in semi-continuous mode, with glucose as the organic carbon source. Chemically induced random mutagenesis was performed to develop a chlorophyll-deficient mutant of *Parachlorella kesslerii*. Both biomasses were aseptically collected, centrifuged, spray-dried, powdered, and stored in airtight containers under controlled conditions until further analysis and use. While detailed cultivation parameters, such as strain identification and specific biomass separation or drying methods, are proprietary and were not fully disclosed by the suppliers, readers interested in these details are encouraged to contact the respective companies (Allmicroalgae and Algaeforfood) directly.

The biochemical composition of the microalgal biomass was analyzed for protein, lipid, fiber, and ash content. Moisture and ash were determined according to ICC methods 110/1 and 104/1, respectively. Lipid extraction followed the method of Bligh and Dyer (1959) [37]. Protein content was measured using the Dumas method (Thermo Quest NA 2100 Nitrogen and Protein Analyzer, Interscience, Breda, The Netherlands), with a nitrogen-to-protein conversion factor of 6.25. Crude fiber was assessed by the Weende method (AOAC method 978.10). Carbohydrate content was calculated by subtracting the protein, lipid, ash, and moisture contents from the total biomass.

### 3.2. Preparation of Oil-in-Water Emulsions

**Figure 7:**
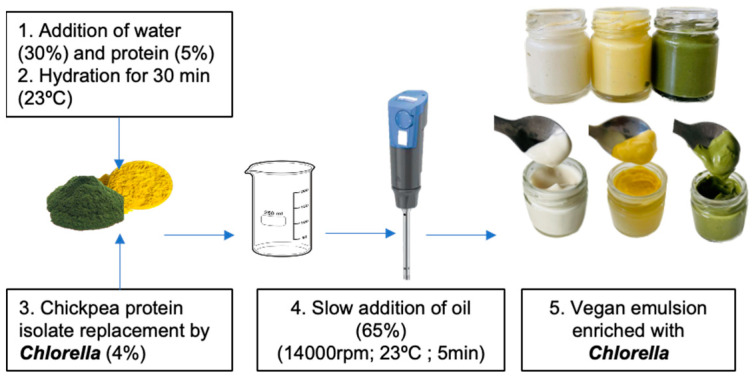
Schematic overview and photographs of the formulation of Auto-*Chlorella* (green) and Hetero-*Chlorella* (yellow) based emulsion.

Batches of 100 g of vegan emulsions were prepared with the following composition: 1% chickpea protein, 4% *Chlorella* biomass, 30% water, and 65% sunflower oil. The control emulsion was prepared using 5% chickpea protein without any microalgae. The process began by dispersing the chickpea protein and microalgae in distilled water using magnetic stirring for 30 min at room temperature (20–22 °C). Once the protein was adequately dispersed and hydrated, sunflower oil (65% *w*/*w*) was introduced under agitation using an Ultra Turrax T-25 homogenizer (IKA, Königswinter, Germany) at 14,000 rpm for 10 min, following the procedure described by [22]. The emulsions were then processed and analyzed for various properties. A detailed illustration of the preparation steps can be found in Figure 7.

The selection of chickpea protein was based on its proven ability to produce stable emulsions, as demonstrated in recent work by Braga and coworkers [4]. Additionally, laboratory-scale experimental trials were conducted to optimize the formulation, particularly to incorporate a higher amount of *Chlorella* biomass (4%), which surpasses levels typically used in prior studies. The aqueous phase consisted of distilled water, and the oil phase utilized sunflower oil at 65% (*w*/*w*), replicating the characteristics of commercial emulsions. This design aimed to balance stability with enhanced nutritional functionality while ensuring reproducibility and scalability.

The primary objective was to develop a formulation that replicated the physical, chemical, and sensory characteristics of a commercial standard sauce while enriching it with a substantial quantity of microalgal biomass. To achieve this goal, a preliminary study (not extensively discussed in the current work) was conducted to optimize emulsion production, where various commercially available microalgal biomasses, such as Honey *Chlorella*, Smooth *Chlorella*, and White *Chlorella* from All microalgae—Natural Products, SA, each with distinct biochemical compositions, were tested. Once the production process was completed, the emulsions were placed in cylindrical glass jars (62 mm in diameter, 56 mm in height) and refrigerated at 4 °C for 24 h to reach equilibrium. Only the emulsions containing whole autotrophic green *Chlorella vulgaris* (Auto-) (Allmicroalgae—Natural Products, Outer West Durban, South Africa) and a yellow *Parachlorella kessleri* (Hetero-*Chlorella*) from Algaeforfood company displayed visual stability without phase separation and were thus retained for further analysis (Figure 7).

### 3.3. Rheology Characterization of the Emulsions

To assess the impact of chickpea protein substitution by microalgal biomasses, Small Amplitude Oscillatory Shear (SAOS) measurements were performed at 20° ± 0.1 °C within a previously determined linear viscoelastic region (LVR), which had been assessed for each sample to obtain the mechanical spectrum. These measurements were taken over a frequency range spanning from 0.01 Hz (0.0628 rad/s) to 100.0 Hz (628 rad/s), using a Haake Mars III controlled-stress rheometer from Thermo Fisher Scientific (Waltham, MA, USA), equipped with a UTC Peltier for temperature control. A serrated parallel plate geometry with a 35 mm diameter (PP35) was employed to minimize slip effects, with a gap of 1.0 mm maintained between the plates.

The mechanical spectrum, represented by the storage modulus (G′) and the loss modulus (G”), as functions of frequency, as well as the loss tangent (tan δ = G”/G′), was obtained. The plateau modulus (G^0^_N_) was determined as the value of G′ corresponding to the minimum point of the loss tangent, as described in previous literature [12,38].

Flow curves were generated within a shear rate range of 10^−5^ to 500 s^−1^ at 20 °C. The Williamson model, as applied by other authors [5,22], was used to fit these curves, using Origin 2019 software (OriginLab Corporation, Northampton, MA, USA).

(1) $$\eta =\frac{\eta_{0}}{1+{\left(k\dot{\gamma }\right)}^{m}}$$

The model Equation (1) includes parameters such as shear rate ($\dot{\gamma }$) (s^−1^), apparent viscosity (η) (Pa.s), zero-shear rate limit viscosity (η_0_), consistency coefficient (k), and a dimensionless shear-thinning index (m). Measurements were conducted a minimum of three times for each formulation.

### 3.4. Droplet Size Distribution of the Emulsions

The droplet size distribution (DSD) of the emulsions was assessed using a laser diffraction instrument (Mastersizer 2000 from Malvern Instruments, Worcestershire, UK) [22]. Prior to measurement, samples were dispersed in distilled water and stirred to ensure uniformity. Two average diameters were calculated: the Sauter mean diameter (d3,2), which represents the average diameter of the majority of droplets, and the De Brouckere Diameter (d4,3). The latter is associated with changes in particle size resulting from various destabilizing mechanisms like droplet aggregation [39]. These parameters were evaluated computationally following Equations (2) and (3), respectively. Additionally, the span parameter (4) was calculated to analyze the profiles of the DSD [40]. A higher span value indicates greater polydispersity amongst droplets, signifying a wider range of droplet sizes within the emulsion, generally associated with lower stability over time [5].

(2) $$d_{3,2}=\frac{\sum n_{i}d_{i}^{3}}{\sum n_{i}d_{i}^{2}}$$

(3) $$d_{4,3}=\frac{\sum n_{i}d_{i}^{4}}{\sum n_{i}d_{i}^{3}}$$

(4) $$Span=\frac{d\left(90\right)-d(10)}{d(50)}$$

where n_i_ is the number of droplets that have d_i_ diameter d_(x)_, corresponding to the x volume percentile of droplets with diameters smaller or equal to these values (10, 50, and 90).

### 3.5. Analysis of Texture Profile

Texture profile analysis (TPA) was conducted using a TAXTplus texturometer from Stable MicroSystems (UK), equipped with a 5 kg load cell. Penetration tests were performed with a cylindrical probe (19 mm diameter) at pre-test, test, and post-test speeds of 1 mm/s, with the samples contained in a cylindrical container (56 mm height and 62 mm diameter). The emulsions were equilibrated at 20 °C for 1 h in a temperature-controlled room prior to the analysis. Firmness (N) was defined as the maximum resistance force during the initial compression cycle, while adhesiveness (−N·s) represented the work needed to separate the probe from the sample. Both firmness and adhesiveness are critical textural parameters closely linked to rheology, making them suitable for characterizing emulsion texture.

### 3.6. pH Monitoring and Color Analysis

A pH-meter (Seven Compact pH-meter S220, Mettler Toledo AG, Greifensee, Switzerland) calibrated with standard buffer solutions (pH 4 and pH 7) was employed to measure the pH of the samples at room temperature (20 °C).

The color of the emulsions was quantitatively assessed using a chromameter (CR400, Minolta, Japan) based on the CIELAB color system parameters, which include L* (brightness, with values increasing from 0 to 100), a* (indicating redness or greenness, with +60 for red and −60 for green), and b* (indicating yellowness or blueness, with +60 for yellowness and −60 for blueness). Measurements were conducted using a D65 standard illuminant and a 2° observer angle under consistent artificial fluorescent lighting conditions. A white standard (L* = 94.61, a* = −0.53, b* = 3.62) at a constant temperature of 20 ± 1 °C.

The total color change (∆E) was calculated for each sample using the following formula:

(5) $$\Delta E^{*}=\sqrt{{{\left(L_{i}^{*}-L_{0}^{*}\right)}^{2}+{\left(a_{i}^{*}-a_{0}^{*}\right)}^{2}+\left(b_{i}^{*}-b_{0}^{*}\right)}^{2}}$$

### 3.7. Antioxidant Potential Assays

The extraction of antioxidant compounds from emulsion samples and the subsequent evaluation of antioxidant activity were performed by adapting the method outlined by [41]. In this modified procedure, 2 g of the sample were mixed with 2 mL of a methanol-water solution (70:30) and 2 mL of hexane, followed by thorough vortex mixing for 1 h. After mixing, the hydrophilic phase was separated from the oil phase using a refrigerated centrifuge apparatus (NF 1200R, Nüve, Ankara, Turkey), operating at 7000 rpm at 4 °C for 10 min. The hydrophilic extracts were then recovered using a syringe, filtered through a 0.25 μm nylon filter with a 15 mm diameter (Thermo Fisher Scientific, Waltham, MA, USA). The lipophilic phase was re-extracted with a methanol-water solution (70:30), and the extracts were treated similarly to the hydrophilic phase. Both extracts were studied for the quantification of phenolic compounds and assessment of antioxidant activity.

Total phenolic content (TPC) was assessed using the Folin–Ciocalteu (FC) method, following the protocol outlined by [10]. In brief, 20 μL of each emulsion extract was combined with 100 μL of FC reagent in a ratio of 1:4 and allowed to settle for 5 min at room temperature. Subsequently, 80 μL of a 7.5% sodium carbonate solution was added to the mixture and thoroughly mixed. After incubating in the dark at room temperature for 2 h, the absorbance was measured at a wavelength of 760 nm using a microplate reader (Thermo Scientific Multiskan GO spectrophotometer from ThermoFisher Scientific). Gallic acid was applied as the standard. The total phenolic content (TPC) in the extract was quantified and expressed in micrograms of gallic acid equivalent (GAE) per gram of emulsion (μgGAEq/g).

The Radical Scavenging Ability (RSA) was assessed utilizing the DPPH (2,2-diphenyl-1-picrylhydrazyl) method, as adapted from the protocol originally developed by [42] and further adjusted by [10]. A total of 20 μL of the extracts were combined with 180 μL of a methanolic DPPH solution with a concentration of 60 μM. This mixture was placed in 96-well microplates. After a 30 min incubation period, the absorbance was measured at a wavelength of 517 nm. The results were expressed as Trolox equivalents per gram (μgTroloxEq/g).

The Ferric Reducing Antioxidant Power (FRAP) assay, proposed by [43], was modified to enable execution in 96-well microplates. To prepare the FRAP solution, 10 mL of acetate buffer (300 mM), pH-adjusted to 3.6 by using acetic acid, was blended with 1 mL of ferric chloride hexahydrate (20 mM) dissolved in distilled water and 1 mL of 2,4,6-Tris(2-pyridyl)-s-triazine (TPTZ) (10 mM) dissolved in HCl (40 mM). Within 96-well microplates, 25 μL of the extract was combined with 175 μL of the FRAP solution (prewarmed to 37 °C) and left to incubate for 30 min in the dark and at room temperature. The results were expressed as Trolox equivalents per gram (μgTroloxEq/g).

### 3.8. Mineral Profile Characterization

The mineral profile was evaluated by exposing the samples to acid digestion. In summary, 0.5 g of emulsion samples were digested in the presence of HNO3 and HCl (3:1) in a heating block (DigiPrep MS, SCP Science, Quebec, QC, Canada) at 105 °C. After the digestion process had concluded and the tubes had been allowed to cool to room temperature, the samples were cautiously transferred, within a fume hood, into 50 mL volumetric flasks. These flasks were then carefully filled to the mark with deionized water, ensuring accurate volume adjustment. The determination of mineral elements was performed by ICP-OES (iCAP 7000 series, Thermo Scientific, Waltham, MA, USA).

### 3.9. Preparation and Quantification of Cobalamin (VitB12)

The preparation of emulsion samples for Vitamin B12 quantification was set in the course of this study. Emulsions were thoroughly mixed with an equal volume of hexane and water, allowing for a 10 min incubation to ensure a clean phase separation. Subsequently, the mixture underwent a 10 min centrifugation process at room temperature (RT, 23 °C) at 10,000 rpm, resulting in the removal of the organic phase. The aqueous phase was precisely defined and completely extracted using a capillary technique. This operation was repeated until 30 mL of the aqueous phase was successfully collected. The collected aqueous phase underwent further centrifugation for 15 min at 10,000 rpm at room temperature and was subsequently passed through a Whatman filter to eliminate any residual solid material. The quantification of Vitamin B12 was then performed based on AOAC Official Method 2014.02 in a sodium acetate buffer (50 mM, from Biochem Chemopharma) containing cyanide (Potassium cyanide 97%, from Across Organic™) at 100 °C for 30 min. Prior to this, an enzymatic hydrolysis (0.05 g α-amylase from porcine pancreas (14 units/mg, SIGMA at 45 °C for 30 min) was performed to efficiently release the bound Vitamin B12. The extracts were further purified and concentrated using an immunoaffinity column (the Easi-Extract^®^ Vitamin B12 (LGE) from R-Biopharm Rhône Ltd., Glasgow, Scotland). The quantification of Vitamin B12 was performed by high-performance liquid chromatography (HPLC), using a Dionex UltiMate 3000 HPLC system, featuring a solvent rack with a 4-channel degasser SRD-3400, a quaternary gradient pump LPG-3400SD, an analytical auto-sampler WPS-3000SL, a column compartment TCC-3000SD, and a UV spectrophotometric detector DAD-3000 operating at 361 nm. The method employed isocratic elution using acetonitrile and trifluoroacetic acid (0.025%) in a 15:85 ratio as the mobile phase, with a flow rate of 0.25 mL/min.

### 3.10. In Vitro Antimicrobial Activity of Microalgae Biomasses

The in vitro antimicrobial activity of the microalgae biomasses incorporated into the emulsion recipe was assessed using the drop testing method [5]. Table 8 outlines the tested microorganisms, sourced from the ISA bacteria library. The capability of contaminant microorganisms to proliferate in the presence of microalgae biomasses was investigated. Bacterial strains were cultivated until reaching the mid-exponential phase. Dilutions ranging from ten-fold to 10^−6^ were prepared in 96-well plates, and 3 μL of each dilution was spotted onto plates containing the relevant medium. In a preliminary test (Test I), microalgae-based media with the same concentrations of microalgae as used in the emulsion recipe (4%) were employed to assess the ability of different microorganisms to thrive in the presence of microalgae biomasses without the presence of a proper nutritive medium. In a subsequent test (Test II), nutritious media were formulated with the same concentrations of microalgae biomass. This second assay aimed to evaluate the in vitro inhibitory capacity, as the inability to grow in an appropriate medium with added microalgae suggested inhibitory potential. All plates were photographed after a 48 h incubation period. Plates with media suitable for each microorganism served as controls, and the entire experimental procedure was conducted in duplicate.

### 3.11. Microbial Stability of Microalgae-Based Emulsions

The microbial stability of the microalgae-based emulsions was assessed both seven days after emulsion production and 60 days later after being stored at 4 °C, as outlined in Table 9.

### 3.12. Statistical Analysis

Origin Pro (version 8.0) software (OriginLab Corporation, Northampton, MA, USA) was used to perform the analysis of variance (one-way ANOVA), using Tukey’s approach for multiple comparisons of means. Results were considered significantly different when *p* < 0.05. All measurements and analyses were performed at least in triplicate, and results are presented as mean values ± standard deviation.

## 4. Conclusions

This study delved into the innovative approach of replacing part of the chickpea protein isolate, used in a vegan emulsion, with microalgae biomass in order to improve the physical properties and nutrition profile. Through a comprehensive analysis, we observed compelling evidence of the protein mimetic capacity of microalgae, potentially influenced by a synergistic interplay between various biomolecules present in the microalgae biomass. Moreover, the emulsifying agents within the microalgae played a pivotal role in enhancing the physical properties of the emulsions, leading to reduced oil droplet size and improved firmness and adhesiveness. Notably, our findings unveiled an attractive color profile alongside enriched levels of antioxidants and dietary elements. Additionally, the rheological behavior and viscosity were significantly impacted by the replacement, exhibiting higher viscoelastic functions (G′, G″, and G^0^_N_) compared to the control, suggesting a more complex three-dimensional structure. Furthermore, the promising detection of Vitamin B12 in the emulsions warrants further exploration into its bioavailability. However, we emphasize the importance of vigilance regarding the microbiological stability of microalgae-based products, considering their nutritional composition, which may serve as a potential food source for bacteria. Although a formal sensory analysis was not performed in the current study, as the primary objective was to evaluate and adjust the physical properties of the emulsions and determine the feasibility of incorporating 4% microalgae (from both autotrophic and heterotrophic modes), future work will include sensory evaluations. These will be crucial in assessing consumer acceptance as we transition to industrial-scale production, particularly for microalgae-based mayonnaise, where stringent preservation measures will be implemented to ensure both safety and sensory satisfaction.

## Figures and Tables

**Table 1:** Comparison of nutritional composition for different microalgal biomasses under various cultivation conditions and the present study.

%	Trophic Mode	Moisture	Protein	Lipids	Carbohydrates	Fiber	Ash	Reference
*Chlorella vulgaris*	Autotrophic	8.30 ± 0.02 ^a^	51.46 ± 0.17 ^a^	10.55 ± 0.66 ^a^	6.60 ± 0.50 ^a^	13.95 ± 1.28 ^a^	9.15 ± 0.06 ^a^	Current study
*P. chlorella kessleri*	Heterotrophic in dark	3.92 ± 0.08 ^b^	49.4 ± 0.19 ^b^	11.18 ± 0.17 ^a^	12.21 ± 2.90 ^b^	18.14 ± 2.57 ^a^	5.15 ± 0.05 ^a^	Current study
*P. chlorella kessleri*	Autotrophic	nd	41.29 ± 0.90 ^c^	20.14 ± 0.58 ^b^	34.15 ± 0.42 ^c^	nd	nd	[15]
*P. chlorella kessleri*	Mixotrophic	nd	44.75 ± 0.26 ^d^	15.45 ± 0.97 ^c^	37.78 ± 0.33 ^c^	nd	nd	[15]
*P. chlorella kessleri*	Heterotrophic in dark	nd	44.15 ± 0.60 ^d^	20.47 ± 0.70 ^b^	35.38 ± 0.21 ^c^	nd	nd	[15]
*Chlorella vulgaris*	Autotrophic	6.2 ± 0.5	41.4 ± 0.4	12.8 ± 0.1	26.7 ± 1.2	5.6 ± 0.4	7.3 ± 0.2	[16]
*Spirulina platensis*	Autotrophic	10.1 ± 0.4	42.8 ± 0.1	5.5 ± 1.2	21.5 ± 0.5	8.5 ± 0.5	11.6 ± 0.3	[16]

nd: Not determined. Different superscripts within the same column indicate significant difference (*p* < 0.05).

**Table 2:** Viscoelastic parameters (G′ and G″ at 1 Hz (6.28 rad/s) and G_0_^N^) from mechanical spectra of microalgae-based emulsions. C-emulsion is the product without *Chlorella* addition.

Samples	G′ at 1 Hz (Pa)	G″ at 1 Hz (Pa)	Plateau Modulus, G^0^_N_ (Pa)
C-emulsion	543.74 ± 20.04 ^c^	55.91 ± 1.75 ^c^	536.34 ± 20.48 ^c^
Hetero-*Chlorella*	926.44 ± 53.94 ^a^	158.45 ± 29.50 ^a^	863.27 ± 90.73 ^a^
Auto-*Chlorella*	811.97 ± 49.71 ^b^	112.98 ± 12.06 ^b^	705.28 ± 60.45 ^b^

Data shown are mean ± SD, *n* = 3. Different superscripts within the same column indicate significant difference (*p* < 0.05).

**Table 3:** Parameters (η_0_, k, and m) from Williamson’s model adjusted to the flow curves of microalgae-based emulsions. C-emulsion is the product without *Chlorella* addition.

Samples	η_0_ (10^3^ Pa.s)	k (10^3^ Pa.s)	m (Dimensionless)
C-emulsion	17.69 ± 1.52 ^c^	0.18 ± 0.06 ^a^	0.97 ± 0.06 ^a^
Hetero-*Chlorella*	30.77 ± 2.09 ^a^	0.17 ± 0.03 ^a^	0.99 ± 0.02 ^a^
Auto-*Chlorella*	25.92 ± 3.50 ^b^	0.19 ± 0.05 ^a^	1.00 ± 0.00 ^a^

Data shown are mean ± SD, *n* = 3. Different superscripts within the same column indicate significant difference (*p* < 0.05).

**Table 4:** The CIELAB color parameters (L*, a*, b*) and pH of microalgae-based emulsions. C-emulsion is the product without *Chlorella* addition.

	C-Emulsion	Hetero-*Chlorella*	Auto-*Chlorella*
T0			
pH	6.83 ± 0.06 ^aA^	6.06 ± 0.06 ^bB^	6.06 ± 0.15 ^bA^
L*	89.55 ± 0.87 ^aA^	75.05 ± 0.01 ^bA^	42.04 ± 0.92 ^cA^
a*	−0.69 ± 0.07 ^aA^	−2.24 ± 0.09 ^bA^	−6.54 ± 0.46 ^cA^
b*	13.9 ± 0.44 ^cA^	30.87 ± 0.37 ^aA^	17.56 ± 1.07 ^bA^
∆E*	_	22.37	48.01
T15			
pH	6.77 ± 0.06 ^aA^	5.80 ± 0.00 ^cB^	6.03 ± 0.00 ^bB^
L*	84.18 ± 0.55 ^aA^	70.18 ± 1.88 ^bA^	47.58 ± 0.61 ^cA^
a*	−0.53 ± 0.02 ^aA^	−2.02 ± 0.05 ^bA^	−3.02 ± 0.54 ^bB^
b*	12.03 ± 0.77 ^bA^	27.53 ± 1.46 ^aA^	11.79 ± 1.69 ^bB^
∆E*	_	18.38	33.71

Data shown are mean ± SD, *n* = 3. Different small superscripts within the same row indicate a significant difference (*p* < 0.05), and different capital superscripts within the same column indicate a significant difference for the same sample within the same parameter after 15 days of preparation.

**Table 5:** Antioxidant parameters in different fractions of microalgae-based emulsion. C-emulsion is the product without Chlorella addition.

		PC	DPPH	FRAP
	Samples	µg GAEq/g	µg TroloxEq/g	µg TroloxEq/g
hydro-alcoholic phase	C-emulsion	44.76 ± 1.38 ^c^	11.20 ± 1.29 ^c^	4.96 ± 0.71 ^c^
	Hetero-*Chlorella*	109.21 ± 1.27 ^a^	85.78 ± 2.05 ^a^	31.38 ± 2.37 ^a^
	Auto-*Chlorella*	87.51 ± 1.02 ^b^	66.51 ± 4.87 ^b^	24.90 ± 4.41 ^b^
Oil phase	C-emulsion	14.29 ± 0.42 ^c^	-	-
	Hetero-*Chlorella*	23.76 ± 1.02 ^a^	-	47.14 ± 0.54 ^a^
	Auto-*Chlorella*	18.31 ± 0.80 ^b^	-	4.70 ± 0.20 ^b^

Data shown are mean ± SD, *n* = 3. Different superscripts within the same row and group indicate a significant difference (*p* < 0.05). - not detected.

**Table 6:** Content of essential dietary elements in microalgae-based emulsions. C-emulsion is the product without *Chlorella* addition.

mg/100 g	C-Emulsion	Hetero-*Chlorella*	Auto-*Chlorella*
K (15% RDV = 300) *	138.04 ± 1.23 ^a^	52.39 ± 0.66 ^c^	73.44 ± 1.08 ^b^
Ca (15% RDV = 120)	4.90 ± 0.44 ^b^	3.29 ± 0.75 ^b^	47.72 ± 0.67 ^a^
Mg (15% RDV = 45)	5.57 ± 0.04 ^b^	11.40 ± 0.68 ^a^	12.27 ± 0.02 ^a^
P (15% RDV = 120)	38.29 ± 0.04 ^c^	76.25 ± 2.80 ^b^	93.79 ± 0.80 ^a^
Fe (15% RDV = 2.1)	1.88 ± 0.05 ^b^	1.29 ± 0.38 ^b^	7.26 ± 0.17 ^a^
Zn (15% RDV = 2.3)	0.34 ± 0.00 ^b^	0.19 ± 0.03 ^b^	1.69 ± 0.86 ^a^
VitB12	n.d	n.d	0.5

Data shown are mean ± SD, *n* = 3. Different superscripts within the same line indicate significant difference (*p* < 0.05). * According to the recommended daily values (RDV) established by Regulation (European Community) No. 1924/2006; Directive No. 90/494 (EC). n.d not detected.

**Table 7:** Evolution of microbial counts in microalgae-based emulsion from day 7 to day 60 under refrigeration. C-emulsion is the product without *Chlorella* addition.

	Samples	Total Mesophilies (10^7^ CFU/mL)	Yeast and Molds (10^3^ CFU/mL)	*E. coli* (10^2^ CFU/mL)	*S.aureus* Coagulase + (10^2^ CFU/mL)	Lactic Acid Bacteria (10^3^ CFU/mL)	*Bacillus cereus* (CFU/mL)	*Listeria monocytogenes* (CFU/mL)	*Salmonella* sp. (CFU/mL)
Day-7	C-emulsion	>300	1000	30	<1	<0.01	<100	<100	<100
	Hetero-*Chlorella* emulsion	210	430	21	<1	<0.01	<100	<100	<100
	Auto-*Chlorella* emulsion	>300	300	>30	<1	<0.01	<100	<100	<100
Day-60	C-emulsion	6.7	270	<1	3.0	>3000	<100	<100	<100
	Hetero-*Chlorella* emulsion	1.9	6.0	<1	180	<1	<100	<100	<100
	Auto-*Chlorella* emulsion	7.4	50	<1	120	>3000	<100	<100	<100

**Table 8:** Pathogenic microorganisms used to test the antimicrobial activity of microalgae biomasses by drop test.

Contaminant	Codes	Growth Conditions
*Listeria innocua*	BISA 3008	Blood Heart Infusion medium (BHI)
*Staphylococcus aurus*	BISA 3966	37 °C
*Escherichia coli*	BISA 3967	
*Salmonella typhimurium*	BISA 3969	
*Bacillus cerreus*	BISA 4043	BHI; 28 °C

**Table 9:** Methodologies used for evaluating the microbial stability of microalgae-based emulsions.

Quantification Methods	Methodology
Total mesophiles	ISO 6610
Total molds and yeasts	NP 3277-1
Total lactic acid bacteria	ISSO 16649-2
*Staphylococcus aureus coagulase+*	ISO 6888
*Escherichia coli*	ISO 16649-2
*Listeria monocytogenes*	ISO 11290
*Salmonella* sp.	ISO 6579

## Data Availability

The data supporting the reported results can be requested by email to the author (Sheymak@isa.ulisboa.pt).
